# 1064. Intra-Host SARS-CoV-2 Evolution Dynamics

**DOI:** 10.1093/ofid/ofac492.905

**Published:** 2022-12-15

**Authors:** Kim El Haddad, Frank Esper, Thamali Madhushani Adhikari Mudiyanselage, Jing Li, Tu Zheng, Yu-Wei Cheng, Daniel D Rhoads, Daniel H Farkas, Jennifer Ko, Sarah Worley, Brian Rubin, Xiangyi Zhang, Xiaoyi Leng

**Affiliations:** Cleveland Clinic Children's, Cleveland, Ohio; Cleveland Clinic Children's, Cleveland, Ohio; Case Western Reserve University, Cleveland, Ohio; Case Western Reserve University, Cleveland, Ohio; Cleveland Clinic Foundation, Cleveland, Ohio; Cleveland Clinic Foundation, Cleveland, Ohio; Cleveland Clinic Foundation, Cleveland, Ohio; Cleveland Clinic Foundation, Cleveland, Ohio; Cleveland Clinic Foundation, Cleveland, Ohio; Cleveland Clinic, Cleveland, OH; Cleveland Clinic Foundation, Cleveland, Ohio; Case Western Reserve University, Cleveland, Ohio; Case Western Reserve University, Cleveland, Ohio

## Abstract

**Background:**

Our understanding of SARS-CoV-2 evolution is limited. Most estimates arise from analysis of global databases populated with unrelated sequences and is currently estimated at ∼27.7 substitutions/genome/year. SARS-CoV-2 polymerase contains a proofreading function encoded by NSP-14 limiting change. Additionally, virus evolution may be influenced by patient comorbidity. Intra-host mutational rate (MR) during infection remain poorly studied.

**Methods:**

To minimize effect of vaccination and/or natural immunity on MR analysis, paired samples from adults originating from the initial pandemic wave (3/17/2020 through 5/27/2020) were retrieved and analyzed at Cleveland Clinic. Viral genome analysis was performed using next generation sequencing, and mutations between paired samples were quantified at allele frequencies (AF) ≥ 0.1, ≥ 0.5 and ≥ 0.75 and compared. MR was calculated employing F81 and JC69 evolution­­­­ models and compared between isolates with (Δ NSP-14) and without (wildtype, wt) non-synonymous mutations in NSP-14 and by comorbidity.

**Results:**

A total of 40 patients (80 sample pairs) were identified. Median interval between paired tests was 15 days [range 5-32]. The estimated MR by F81 modeling was 317.2 (95%CI 312.0-322.3), 54.6 (95%CI 52.5-56.7) and 45.1 (95%CI 43.1-47.0) substitutions/genome/year at AF of ≥0.1, ≥0.5, ≥0.75 respectively. Rates in ΔNSP-14 (n=13) vs wt (n=27) groups were 557.7 (95%CI 537.0-578.2) vs 193.1 (95%CI 187.1-199.1) p-value 0.001, 50.8 (95%CI 44.3-57.3) vs 56.3 (95%CI 53.1-59.4) p-value 0.144, and 31.0 (95%CI 25.9-36.1) vs 51.3 (95%CI 48.3-54.3) p- value < 0.001 at AF ≥0.1, ≥0.5, ≥0.75 respectively. Patients with immune comorbidities (n=6) had significantly higher MR of 137.6 (95%CI 114.6-160.5) vs 40.5 (95%CI 38.4-42.7) p-value < 0.001, and 113.7 (95%CI 92.8-134.5) vs 33.4 (95%CI 31.5-35.4) p-value < 0.001 at AF ≥0.5 and ≥0.75 respectively. Similar results were obtained when using the JC69 model.

**Figure:**
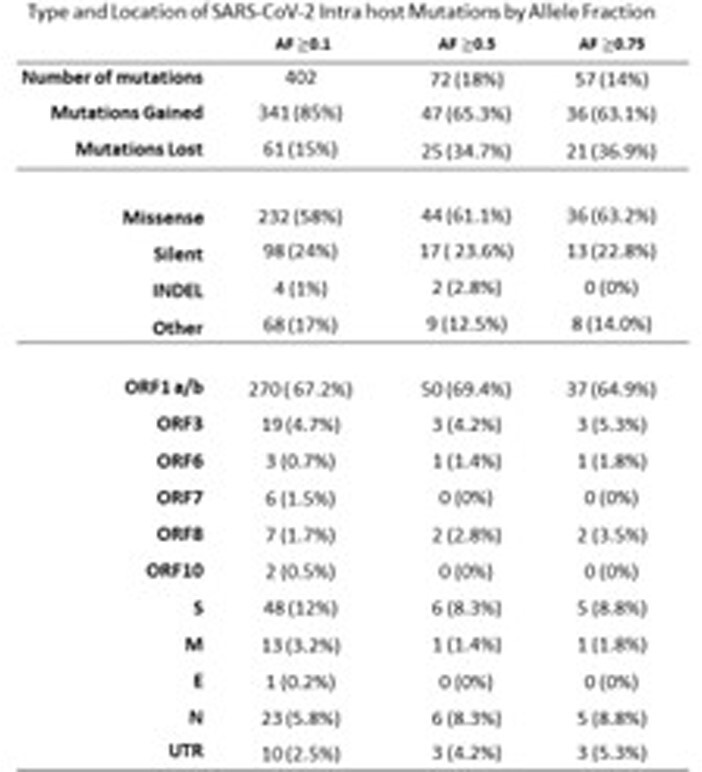

**Figure:**
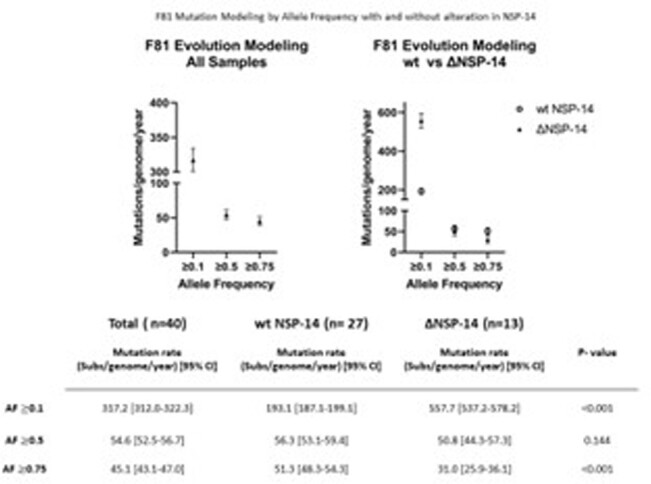

**Figure:**
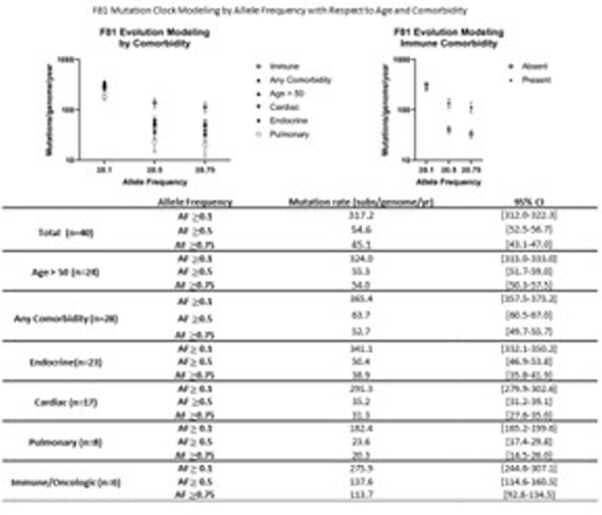

**Conclusion:**

Intra-host SARS-CoV-2 mutation rates are higher than those reported through population analysis. Virus strains with altered NSP-14 have accelerated MR at low AF. Immunosuppressed patients have elevated MR at higher AF. Understanding intra-host virus evolution will aid in current and future pandemic modeling.

**Disclosures:**

**Frank Esper, M.D**, Johnson and Johnson: Advisor/Consultant **Daniel D. Rhoads, M.D. PhD**, Luminex: Advisor/Consultant|Talis Biomedical: Advisor/Consultant|Thermo Fisher: Advisor/Consultant.

